# Duodenal Lipoma as a Rare Cause of Upper Gastrointestinal Bleeding

**DOI:** 10.4021/gr260w

**Published:** 2010-11-20

**Authors:** Helga M. Ouwerkerk, Raber, G. Freling, J.M. Klaase

**Affiliations:** a Department of General Surgery, Medisch Spectrum Twente, Enschede, the Netherlands; b Department of Pathology, Medisch Spectrum Twente, Enschede, the Netherlands

**Keywords:** Gastrointestinal, Duodenal lipoma, Melaena

## Abstract

A 52-year-old female was referred because of melaena. After initital work-up, including gastroduodenoscopy, endosonography and CT scan, a duodenotomy was performed. Definite diagnosis was a duodenal lipoma based on histological findings. Lipomas of the gastrointestinal tract are rare. Only 4% occur in the duodenum. The peak incidence is around the 5th and 7th decade of life, with a slight female preponderance. Gastrointestinal lipomas are usually asymptomatic, but can present with mild to severe gastrointestinal bleeding, intussusceptions, abdominal pain, constipation and diarrhea. Clinical, endoscopical, surgical, and radiological features are described in this case of duodenal lipoma.

## Introduction

Lipoma arises from adipocytes. Lipomas are benign, slow growing tumors, and are generally found in subcutaneous tissue of the proximal extremities and trunk. The gastrointestinal (GI) tract is an uncommon localization for lipoma, but if found, their most common localization is the colon. Generally they occur as a single entity, but they may be multiple. Ninety-percent of the gastrointestinal lipomas are located in the submucosa, 10% arise from the subserosa [1]. The shape is variable and they can be either sessile or pedunculated. Gastrointestinal lipomas are usually asymptomatic. We present a 52-year-old woman with a duodenal lipoma manifesting with melaena.

## Case Report

**Figure 1:**
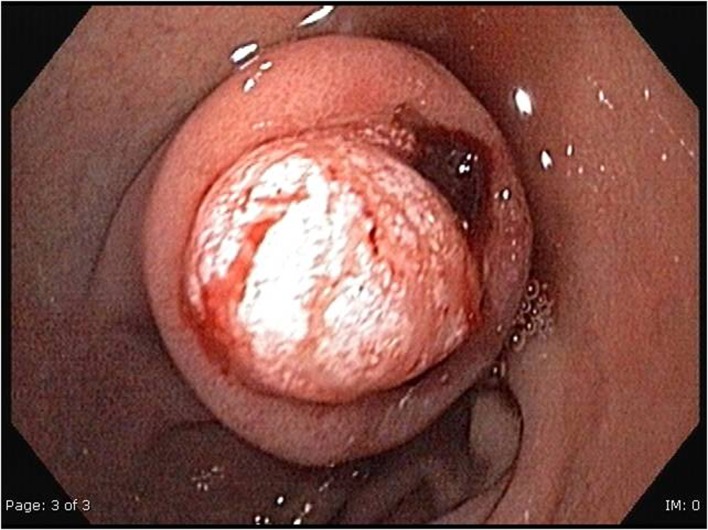
Gastroscopy shows a submucosal tumor of the bulbus duodeni of 1.0 x 2.0 cm in diameter, with a central bleeding stigma.

**Figure 2:**
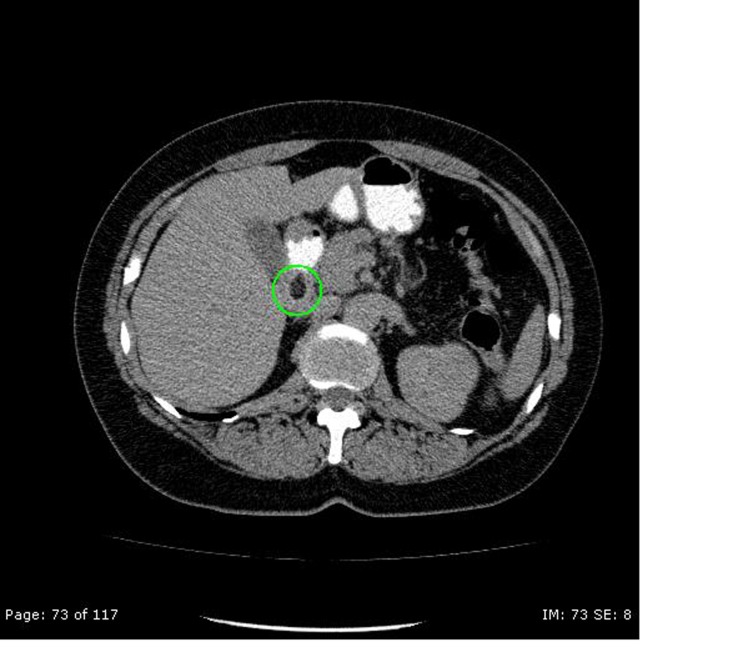
CT of the abdomen shows a T1-weighted image with an overall hypodense homogeneous low signal (-41 Hounsfield unit) lesion.

A 52-year-old woman was admitted to our hospital because of melaena existing for one day, abdominal distension and vomitus. Anamnestic, there were no other complaints. There were no signs of hemodynamic shock. Physical examination showed abdominal distension but no other abnormalities. Her hemoglobin level was 5.7 mmol/l (9.18 g/dL). Gastroscopy revealed a submucosal tumor of the bulbus duodeni of 1.0 x 2.0 cm in diameter, with a central bleeding stigma (Fig. 1). Endoscopic ultrasonography (EUS) showed a hypo-echogenic lesion in the bulbus duodeni, but the intestinal layer of origin remained unknown. The differential diagnosis was gastro-intestinal stromal tumor (GIST) and lipoma. Biopsies taken during the EUS were inconclusive. Computed Tomography of the abdomen revealed a hypodense lesion with the density of fat (-41 Hounsfield unit), leading to the diagnosis of intraluminal lipoma of the duodenum (Fig. 2).

**Figure 3:**
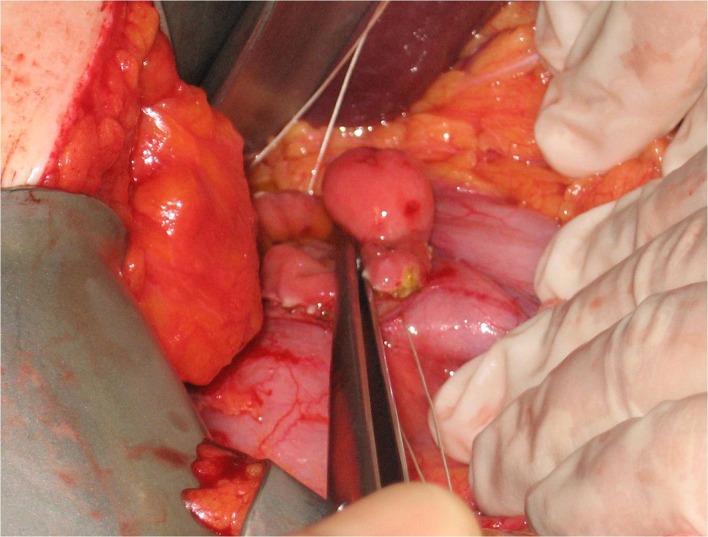
Macroscopic inspection during surgery shows a tumor with a diameter of 2.0 x 1.5 cm, 2 cm distal from the pylorus.

**Figure 4:**
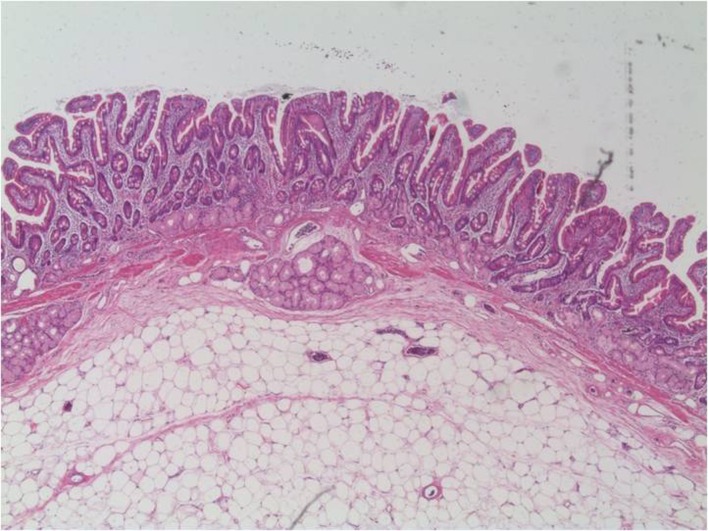
Microscopy shows a lipoma under a normal duodenal mucosal layer.

**Figure 5:**
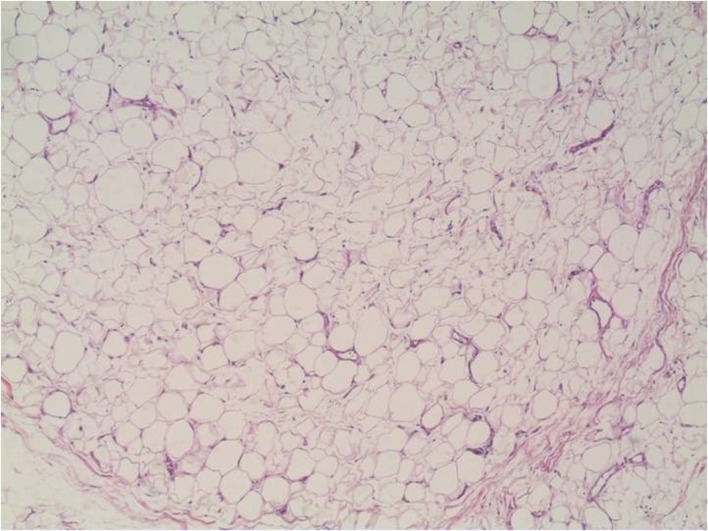
Microscopic image of the lipoma containing mature fat-tissue.

The patient underwent a duodenotomie 28 days after initial presentation. The lipoma was resected (Fig. 3). The postoperative period was uneventful. The patient was discharged home on the fifth postoperative day. Macroscopic inspection at the pathology department showed a pink-orange colored, soft, elastic, and lobulated tumor with a size of 1.7 x 1.5 cm. Microscopy revealed a normal duodenal mucosal layer. The muscularis mucosa was missing in some places. In the underlying stroma, mature fat-tissue was seen with reactive fibrotic septa. No lipoblasts, hyperchromatic nuclei, or reticular vascular network was seen (Fig. 4, 5). These features are characteristic for a duodenal lipoma.

## Discussion

Lipomas are benign, slow growing tumors. In the gastrointestinal (GI) tract they are uncommonly found. Gastrointestinal lipomas account for 4% of all benign gastrointestinal tumors. The most common localization is the colon (64%). Lipomas of the small intestine comprise 17.5% of all benign neoplasm of the small intestine [2]. Of all lipomas of the gastrointestinal tract, only 4% occur in the duodenum [3]. The peak of incidence seems to be around the 5th and 7th decade of life. Gastrointestinal lipomas are usually asymptomatic.

If a duodenal lipoma is symptomatic, the most common findings are ulceration, gastrointestinal bleeding, intussusceptions, or bowel obstruction. The differentiation between a duodenal lipoma and other gastrointestinal tumors, like GIST or liposarcomas, can be made by CT or MRI [4]. On CT, duodenal lipomas appear as a smooth-margined mass with a low Hounsfield unit (range -70 and -120) corresponding with the density of fat [5, 6]. Sometimes a few thin septations can be seen. Intestinal lipomas are distinguishable from liposarcomas by their homogeneity and by the absence of areas of increased density. As for MRI, the signals are low on T1- and T2-weighted fat-suppressed images which is specific for lipoma. Lipoma shows no contrast enhancement.

Endoscopic ultrasonography usually shows a homogenous, hyperechoic mass within the submucosal layer which is highly characteristic [7].

With the evolution of gastrointestinal endoscopy, this tool can be used for diagnosis and treatment, including hemostasis. Duodenal lipoma can be pedunculated or sessile. The pedunculated lipoma can be easily and safely removed by electro surgical endoscopic snare polypectomy [8-10]. However, sessile and big lipomas are more safely removed surgically, as was the case in our patient, since the bigger the lipoma, the higher the risk of hemorrhage and perforation.

In conclusion, duodenal lipoma is an uncommon type of gastro-intestinal neoplasm. It is usually asymptomatic, but can present with (severe) gastrointestinal bleeding, intussusceptions or bowel obstruction. CT and MRI are highly accurate diagnostic tools, and should play an important role in treatment planning. Since lipomas are benign tumors, and no malignant transformation has ever been reported, it might be more desirable to employ the less invasive endoscopic snare polypectomy instead of extended surgical resection whenever this is possible. Moreover intervention should only be performed when the lesion becomes symptomatic (risk-benefit-weight). The definite diagnosis should always be confirmed histologically.
